# Supplementary material for: Diagnostic performance of the WHO definition of probable dengue within the first 5 days of symptoms on Reunion Island
Source: PLoS One. 2024 Feb 15;19(2):e0295260. doi: 10.1371/journal.pone.0295260 (PMC10868786; doi:10.1371/journal.pone.0295260)
Supplement: S1 Table — (DOCX) [file pone.0295260.s001.docx]

**S1Table. Description of the study population on variables associated with RT-PCR.**

| **Characteristic** | **Overall, N = 1,181^a^** | **RT-PCR Negative, N = 491^a^** | **RT-PCR Positive, N = 690^a^** | **p-value^b^** |
| --- | --- | --- | --- | --- |
| **Male** | 632 (54%) | 246 (50%) | 386 (56%) | 0.047 |
| **Age (year)** | 46.43 (24.76, 69.08) | 40.32 (16.70, 68.38) | 50.03 (27.93, 70.02) | <0.001 |
| **Asthenia** | 800 (68%) | 314 (64%) | 486 (70%) | 0.019 |
| **Headache** | 592 (50%) | 222 (45%) | 370 (54%) | 0.004 |
| **Retro-orbital pain** | 227 (19%) | 81 (16%) | 146 (21%) | 0.045 |
| **Myalgia** | 609 (52%) | 222 (45%) | 387 (56%) | <0.001 |
| **Arthralgia** | 411 (35%) | 125 (25%) | 286 (41%) | <0.001 |
| **Backache** | 180 (15%) | 48 (9.8%) | 132 (19%) | <0.001 |
| **Maculo-papular / erythematous rash (MPR)** | 159 (13%) | 49 (10.0%) | 110 (16%) | 0.003 |
| **MPR face** | 33 (2.8%) | 8 (1.6%) | 25 (3.6%) | 0.040 |
| **MPR back** | 60 (5.1%) | 16 (3.3%) | 44 (6.4%) | 0.016 |
| **MPR lower limbs** | 48 (4.1%) | 12 (2.4%) | 36 (5.2%) | 0.017 |
| **Mucocutaneous signs (MCS) lip/cheilitis** | 25 (2.1%) | 3 (0.6%) | 22 (3.2%) | 0.002 |
| **MCS tongue** | 11 (0.9%) | 0 (0%) | 11 (1.6%) | 0.004 |
| **Pharyngitis** | 55 (4.7%) | 35 (7.1%) | 20 (2.9%) | <0.001 |
| **Dysgeusia** | 70 (5.9%) | 16 (3.3%) | 54 (7.8%) | 0.001 |
| **Neurological signs** | 267 (23%) | 86 (18%) | 181 (26%) | <0.001 |
| **Dizziness** | 94 (8.0%) | 27 (5.5%) | 67 (9.7%) | 0.008 |
| **Paresthesia** | 25 (2.1%) | 5 (1.0%) | 20 (2.9%) | 0.027 |
| **Anxiety** | 12 (1.0%) | 1 (0.2%) | 11 (1.6%) | 0.019 |
| **Diarrhea** | 213 (18%) | 72 (15%) | 141 (20%) | 0.011 |
| **Other digestive manifestations** | 49 (4.1%) | 10 (2.0%) | 39 (5.7%) | 0.002 |
| **Cough** | 94 (8.0%) | 49 (10.0%) | 45 (6.5%) | 0.030 |
| **Pleural effusion** | 8 (0.7%) | 7 (1.4%) | 1 (0.1%) | 0.011 |
| **Bleeding** | 147 (12%) | 37 (7.5%) | 110 (16%) | <0.001 |
| **Gingival bleeding** | 34 (2.9%) | 8 (1.6%) | 26 (3.8%) | 0.030 |
| **Hematuria** | 25 (2.1%) | 2 (0.4%) | 23 (3.3%) | <0.001 |
| **Consultation within 48 hours** | 122 (10%) | 22 (4.5%) | 100 (14%) | <0.001 |
| **Red blood cell count (×1,000 G/L)** | 4.60 (4.30, 5.00) | 4.70 (4.30, 5.00) | 4.60 (4.20, 5.00) | 0.045 |
| **Mean Corpuscular Volume of red blood cells (fL)** | 85.40 (81.40, 88.80) | 84.70 (80.10, 88.70) | 85.70 (82.00, 88.90) | 0.026 |
| **White blood cell count (Giga/L)** | 4.90 (3.20, 7.40) | 6.05 (3.48, 9.53) | 4.50 (3.10, 6.30) | <0.001 |
| **Lymphocyte count (Giga/L)** | 0.70 (0.50, 1.12) | 1.00 (0.60, 1.60) | 0.60 (0.40, 0.90) | <0.001 |
| **Lymphocytes (%)** | 8.95 (4.81, 14.04) | 10.30 (5.57, 14.76) | 8.12 (4.66, 13.25) | <0.001 |
| **Monocytes (%)** | 5.65 (4.05, 7.80) | 5.07 (3.60, 6.76) | 6.03 (4.26, 8.25) | <0.001 |
| **Basophil count (Giga/L)** | 0.00 (0.00, 0.00) | 0.00 (0.00, 0.00) | 0.00 (0.00, 0.00) | 0.031 |
| **Eosinophil count (Giga/L)** | 0.00 (0.00, 0.00) | 0.00 (0.00, 0.10) | 0.00 (0.00, 0.00) | <0.001 |
| **Eosinophils (%)** | 0.00 (0.00, 0.45) | 0.10 (0.00, 0.51) | 0.00 (0.00, 0.35) | <0.001 |
| **Platelet count (G/L)** | 170.00 (123.00, 226.00) | 176.00 (123.75, 244.50) | 166.00 (123.00, 214.00) | 0.007 |
| **Neutrophil count (G/L)** | 3.30 (1.80, 5.50) | 4.00 (2.00, 7.10) | 3.00 (1.80, 4.68) | <0.001 |
| **Platelet count (G/L)** | 170.00 (123.00, 226.00) | 176.00 (123.75, 244.50) | 166.00 (123.00, 214.00) | 0.007 |
| **Serum urea (mmol/L)** | 4,500.00 (3,300.00, 6,500.00) | 4,600.00 (3,500.00, 6,925.00) | 4,400.00 (3,200.00, 6,100.00) | 0.015 |
| **Serum Sodium (mmol/L)** | 137.00 (135.00, 139.00) | 137.00 (135.00, 139.00) | 137.00 (135.00, 138.00) | 0.014 |
| **Number of symptoms of 2009 WHO probable dengue** | 2.00 (1.00, 3.00) | 2.00 (1.00, 2.00) | 2.00 (1.00, 3.00) | <0.001 |
| **2009 WHO probable dengue** | 652 (55%) | 213 (43%) | 439 (64%) | <0.001 |

^a^Median (1st quartile, 3rd quartile), count (%).

^b^Wilcoxon rank sum test, Fisher's exact test, Pearson's Chi-squared test.

RT-PCR, Reverse Transcriptase Polymerase Chain Reaction; WHO, World Health Organization.
