# Supplementary material for: Diagnostic performance of the WHO definition of probable dengue within the first 5 days of symptoms on Reunion Island
Source: PLoS One. 2024 Feb 15;19(2):e0295260. doi: 10.1371/journal.pone.0295260 (PMC10868786; doi:10.1371/journal.pone.0295260)
Supplement: Attachment — Submitted filename: Response to Reviewers.pdf [file pone.0295260.s003.pdf]

PONE-D-23-10419
Diagnostic performance of the WHO definition of probable dengue on Reunion Island
PLOS ONE

Dear Dr. Diarra,
Thank you for submitting your manuscript to PLOS ONE. After careful consideration, we feel that it has merit but does not fully meet PLOS ONE's publication criteria as it currently stands. Therefore, we invite you to submit a revised version of the manuscript that addresses the points raised during the review process.

==============================
Both reviewers had some very helpful suggestions for improving the manuscript. It is my hope that the revision process will proceed much faster now that we have identified reviewers.
==============================

Please submit your revised manuscript by Oct 27 2023 11:59PM. If you will need more time than this to complete your revisions, please reply to this message or contact the journal office at plosone@plos.org. When you're ready to submit your revision, log on to https://www.editorialmanager.com/pone/ and select the 'Submissions Needing Revision' folder to locate your manuscript file.
Please include the following items when submitting your revised manuscript:

- A rebuttal letter that responds to each point raised by the academic editor and reviewer(s). You should upload this letter as a separate file labeled 'Response to Reviewers'.
- A marked-up copy of your manuscript that highlights changes made to the original version. You should upload this as a separate file labeled 'Revised Manuscript with Track Changes'.
- An unmarked version of your revised paper without tracked changes. You should upload this as a separate file labeled 'Manuscript'.

If you would like to make changes to your financial disclosure, please include your updated statement in your cover letter. Guidelines for resubmitting your figure files are available below the reviewer comments at the end of this letter.

If applicable, we recommend that you deposit your laboratory protocols in protocols.io to enhance the reproducibility of your results. Protocols.io assigns your protocol its own identifier (DOI) so that it can be cited independently in the future. For instructions see: https://journals.plos.org/plosone/s/submission-guidelines#loc-laboratory-protocols. Additionally, PLOS ONE offers an option for publishing peer-reviewed Lab Protocol articles, which describe protocols hosted on protocols.io. Read more information on sharing protocols at https://plos.org/protocols?utm_medium=editorial-email&utm_source=authorletters&utm_campaign=protocols.

We look forward to receiving your revised manuscript.
Kind regards,
Kelli L. Barr, Ph.D.
Academic Editor
PLOS ONE
Journal Requirements:
When submitting your revision, we need you to address these additional requirements.
1. Please ensure that your manuscript meets PLOS ONE's style requirements, including those for file naming. The PLOS ONE style templates can be found at
https://journals.plos.org/plosone/s/file?id=wjVg/PLOSOne_formatting_sample_main_body.pdf and
https://journals.plos.org/plosone/s/file?id=ba62/PLOSOne_formatting_sample_title_authors_affiliations.pdf
2. Thank you for stating the following financial disclosure:
"Funding was obtained by European Regional Development Fund (RUNDENG 20201640-00222937)."
Please state what role the funders took in the study. If the funders had no role, please state: "The funders had no role in study design, data collection and analysis, decision to publish, or preparation of the manuscript."
If this statement is not correct you must amend it as needed.

Please include this amended Role of Funder statement in your cover letter; we will change the online submission form on your behalf.
3. Thank you for stating the following in your Competing Interests section:
"None to declare."
Please complete your Competing Interests on the online submission form to state any Competing Interests. If you have no competing interests, please state "The authors have declared that no competing interests exist.", as detailed online in our guide for authors at http://journals.plos.org/plosone/s/submit-now
This information should be included in your cover letter; we will change the online submission form on your behalf.
4. We note that you have indicated that data from this study are available upon request. PLOS only allows data to be available upon request if there are legal or ethical restrictions on sharing data publicly. For more information on unacceptable data access restrictions, please see http://journals.plos.org/plosone/s/data-availability#loc-unacceptable-data-access-restrictions.
In your revised cover letter, please address the following prompts:
a) If there are ethical or legal restrictions on sharing a de-identified data set, please explain them in detail (e.g., data contain potentially sensitive information, data are owned by a third-party organization, etc.) and who has imposed them (e.g., an ethics committee). Please also provide contact information for a data access committee, ethics committee, or other institutional body to which data requests may be sent.
b) If there are no restrictions, please upload the minimal anonymized data set necessary to replicate your study findings as either Supporting Information files or to a stable, public repository and provide us with the relevant URLs, DOIs, or accession numbers. For a list of acceptable repositories, please see http://journals.plos.org/plosone/s/data-availability#loc-recommended-repositories.
We will update your Data Availability statement on your behalf to reflect the information you provide.
5. Please include captions for your Supporting Information files at the end of your manuscript, and update any in-text citations to match accordingly. Please see our Supporting Information guidelines for more information: http://journals.plos.org/plosone/s/supporting-information.
6. Please review your reference list to ensure that it is complete and correct. If you have cited papers that have been retracted, please include the rationale for doing so in the manuscript text, or remove these references and replace them with relevant current references. Any changes to the reference list should be mentioned in the rebuttal letter that accompanies your revised manuscript. If you need to cite a retracted article, indicate the article's retracted status in the References list and also include a citation and full reference for the retraction notice.
[Note: HTML markup is below. Please do not edit.]

We are indebted and thankful of Editor's recommendations and guidance for improving our manuscript.

Reviewers' comments:

Reviewer's Responses to Questions

Comments to the Author
1. Is the manuscript technically sound, and do the data support the conclusions?
The manuscript must describe a technically sound piece of scientific research with data that supports the conclusions. Experiments must have been conducted rigorously, with appropriate controls, replication, and sample sizes. The conclusions must be drawn appropriately based on the data presented.

Reviewer #1: Yes

Reviewer #2: Yes

---

2. Has the statistical analysis been performed appropriately and rigorously?

Reviewer #1: Yes

Reviewer #2: Yes

3. Have the authors made all data underlying the findings in their manuscript fully available?

The PLOS Data policy requires authors to make all data underlying the findings described in their manuscript fully available without restriction, with rare exception (please refer to the Data Availability Statement in the manuscript PDF file). The data should be provided as part of the manuscript or its supporting information, or deposited to a public repository. For example, in addition to summary statistics, the data points behind means, medians and variance measures should be available. If there are restrictions on publicly sharing data—e.g. participant privacy or use of data from a third party—those must be specified.

Reviewer #1: Yes

Reviewer #2: Yes

4. Is the manuscript presented in an intelligible fashion and written in standard English?

PLOS ONE does not copyedit accepted manuscripts, so the language in submitted articles must be clear, correct, and unambiguous. Any typographical or grammatical errors should be corrected at revision, so please note any specific errors here.

Reviewer #1: Yes

Reviewer #2: Yes

We are indebted of both reviewers for their very positive assessment of our manuscript and wish to thank them not to have raised major flaws in the four abovementioned bullet questions.

5. Review Comments to the Author

Please use the space provided to explain your answers to the questions above. You may also include additional comments for the author, including concerns about dual publication, research ethics, or publication ethics. (Please upload your review as an attachment if it exceeds 20,000 characters)

We would like to thank the reviewer #1 for his/her overall assessment and his/her pertinent comments, which helped us to improve our manuscript.

We would like to thank Dr. Nujum, the reviewer #2, for her overall assessment and her pertinent comments, which helped us to improve our manuscript

General comments

Reviewer #1: This manuscript provides useful computational information that can positively impact determining probable Dengue within the population on Reunion Island based on clinical serology characteristics. Of which, the results could be utilized with regard to patient diagnosis and management of dengue within this region.

Thanks for this positive comment.

Introduction:

I would like to see a more thorough introduction by providing clinical information on Dengue as it relates to the improvements in question in this study (i.e. model addresses lymphopenia as a factor for probable Dengue – it would be nice to demonstrate the current knowledge in the intro regarding clinical and serological presentation with regards to individuals with dengue virus).

The clinical and biological features currently known to identify probable dengue fever have been added to the introduction to the new version of the manuscript (page 4, lines 82-88). The insert is as follows :

“According to this definition, a patient was suspected of DF and could be diagnosed as probable dengue if he had lived in or traveled to a dengue-endemic area and had fever, plus at least two of the following criteria: nausea or vomiting, rash, aches and pains, positive tourniquet test, leukopenia and any warning sign (abdominal pain or tenderness, persistent vomiting, clinical fluid accumulation, mucosal bleed, lethargy or restlessness, liver enlargement >2 cm, increase in hematocrit concurrent with rapid decrease in platelet count)”

Line 62 – Consider the addition of vector information and how a changing climate you described can impact Dengue expansion in humans

This observation was taken into account. We did it in three sentences page 3, lines 52-61. “Furthermore, regions at risk of dengue are expanding owing to global warming, and DENV transmission is no longer limited to tropical or subtropical areas; it now threatens temperate areas during hot seasons through the adaptation and resilience to climate change of Aedes mosquito vectors (Ae. Albopictus and Ae. Aegypti) [2,3]. Indeed, the multiplication of Aedes vectors and arbovirus pathogens (including DENV) is mainly driven by temperature, which under the pattern of climate change, hastens mosquito reproduction, survival and shortens the extrinsic incubation period, while increasing virus replication [4]. In addition, the photoperiodic diapause of Ae. Albopictus, a mechanism of overwinter survivorship under cold and dark conditions, may increase its seasonal abundance and boost its dynamics of invasion in the Northern hemisphere [5].”

Line 65-67 - Please provide references

Two references [4] and [5] are now given (page 3, lines 56-61).

Line 67 – Rephrase sentence for clarity, consider changing to “before re-emerging” or “before it re-emerged”

Thank you for these suggestions, we have now used "before re-emerging" (page 3, line 64).

Line 70-71 – References

The reference is now given (page 3, line 69).

Line 76 – 78 – References

This comment is now taken into account (page 4, line 75).

Line 85 – Please explain the WHO definition for probable dengue between the sentence “Probable Dengue in 2009” and “However, the relevance….” And remove it from the methods section.

These recommendations are now taken into account (page 4, lines 82-88).

Methods:

Line 102-107 – Remove the WHO 2009 definition from methods section and place in introduction.

Already taken into account in previous recommendations (page 4, lines 82-88).

Line 102 Please replace the WHO definition with the actual particulars of the study participants (i.e. Study population was selected from 2365 participants and consisted of 1662 subjects that were generated from patient data from the ED based on clinical criteria associated with probable Dengue as defined by WHO…..)

These observations are now taken into account in the new version of the manuscript (page 5, lines 98-107). “This retrospective diagnostic study of medical records and export of biomedical laboratory results involved the 1662 subjects admitted to the emergency department (ED) of the University Hospital of Reunion Island between 1st of January and 30th of June 2019 and meeting the inclusion criteria. Inclusion criteria were: suspected DF, maximum 5 days between reported onset of symptoms, admission and RT-PCR result. Subjects were suspected of having DF when they presented with fever and one of the following symptoms and signs: headache, retro-orbital pain, myalgia, arthralgia, rash, or pruritus. These 1662 subjects to whom the 2009 WHO definition of probable dengue was applied were taken from the 2365 subjects suspected of having DF. Depending on the inclusion period, a RDT and/or RT-PCR was performed to confirm the diagnosis in subjects suspected of having DF.”

Results:

Line 151 – Briefly explain the initial exclusion of the remaining 2365 that were not applicable in the study.

This recommendation is taken into account in the new version at the method level (page 5, line 102-104). Indeed, this clarification was also requested by the other reviewer with regard to the study population. “Subjects were suspected of having DF when they presented with fever and one of the following symptoms and signs: headache, retro-orbital pain, myalgia, arthralgia, rash, or pruritus.”

Discussion & Conclusion:

Both Discussion and Conclusion sections are concisely written and no changes are recommended.

Please carefully review the entire manuscript and ensure that all appropriate in-text citations are present, as there appeared to be a few missing references throughout the introduction.

We thank the reviewer for this valuable advice.

Reviewer #2: Diagnostic performance of the WHO definition of probable dengue on Reunion Island

General comment

This is a good study, well done

Thanks for this positive comment.

Specific comments

Title - Would it be better to add that the study included on dengue suspects presenting within 5 days of onset of fever?

Yes, this information can be useful, but to avoid having too long a title, we have added in the new version that the time elapsed between the onset of symptoms and admission. “Diagnostic performance of the WHO definition of probable dengue within the first 5 days of symptoms on Reunion Island”

Study population - It would be good to state the definition of suspect dengue fever, who form the study population and on whom the definition of probable dengue was applied.
It will also be nice to write something about the mechanisms in place for obtaining quality data from records, since it is a retrospective study. What is the protocol in the hospital ? do all suspect cases of dengue fever undergo RT-PCR?

These observations are now taken into account in the new version of the manuscript (page 5, lines 98-111). “This retrospective diagnostic study of medical records and export of biomedical laboratory results involved the 1662 subjects admitted to the emergency department (ED) of the University Hospital of Reunion Island between 1st of January and 30th of June 2019 and meeting the inclusion criteria. Inclusion criteria were: suspected DF, maximum 5 days between reported onset of symptoms, admission and RT-PCR result. Subjects were suspected of having DF when they presented with fever and one of the following symptoms and signs: headache, retro-orbital pain, myalgia, arthralgia, rash, or pruritus. These 1662 subjects to whom the 2009 WHO definition of probable dengue was applied were taken from the 2365 subjects suspected of having DF. Depending on the inclusion period, a RDT and/or RT-PCR was performed to confirm the diagnosis in subjects suspected of having DF.
The data retrieved were sociodemographic, clinical, and biological characteristics. To improve data quality, a data entry guide was used to retrieve sociodemographic and clinical information from patient records. The biological data extracted directly from the laboratory was then merged with the sociodemographic and clinical data.”

Table 1 : Coulumn 3, Row 1 RTPCR Negative , N= 49 is what is written, please check the number , I think last digit is missing (?491)

Thank you for detecting this typing error, which has now been corrected; yes, it is 491.

Discussion - Some points on relevance of using case definitions for dengue surveillance can be added and how the improvement of the case definitions helps to improve the quality of surveillance. Best wishes to the authors.

This point has now been taken into account in the new document (page 12, line 243-256). “The 2009 WHO definition of probable dengue detected 64 out of 100 infected individuals. The remaining 36 would not be identified, which might have a negative impact on patient management and control of the epidemic. The addition of lymphopenia detected 74 out of 100 infected patients, which represents a significant improvement in patient care and makes it easier to quarantine patients so as not to infect other mosquitoes and subsequently other individuals. Furthermore, in the context of an epidemic, when household transmission is confirmed, it is easy to presume DF on the presence of clinical signs among other household members or among people living in the close neighborhood. The addition of the biological indicator (lymphopenia) may be useful in areas where RT-PCR is not readily available, but this addition also implies that blood has been collected, and in countries where molecular testing is readily available, this could have already included the request for a RT-PCR. Regardless of this, as the technique generally provides an answer within 24 to 72 hours, all additional means aimed at guiding the general practitioner or the ED physician in his management of the dengue patient remain legitimate and should be advocated in our health system.”

6. PLOS authors have the option to publish the peer review history of their article (what does this mean?). If published, this will include your full peer review and any attached files.

If you choose “no”, your identity will remain anonymous but your review may still be made public.

Do you want your identity to be public for this peer review? For information about this choice, including consent withdrawal, please see our Privacy Policy.

Reviewer #1: No

Reviewer #2: **Yes:** Zinia Thajudeen Nujum
