# Supplementary material for: Diagnostic performance of the WHO definition of probable dengue within the first 5 days of symptoms on Reunion Island
Source: PLoS One. 2024 Feb 15;19(2):e0295260. doi: 10.1371/journal.pone.0295260 (PMC10868786; doi:10.1371/journal.pone.0295260)
Supplement: Attachment — Submitted filename: PLOS one Dengue.docx [file pone.0295260.s002.docx]

**Diagnostic performance of the WHO definition of probable dengue on Reunion Island**

**General comments**

This is a good study, well done

**Specific comments**

Title - Would it be better to add that the study included on dengue suspects presenting within 5 days of onset of fever?

Study population - It would be good to state the definition of suspect dengue fever, who form the study population and on whom the definition of probable dengue was applied.

It will also be nice to write something about the mechanisms in place for obtaining quality data from records, since it is a retrospective study. What is the protocol in the hospital ? do all suspect cases of dengue fever undergo RT-PCR?

Table 1 : Coulumn 3, Row 1 RTPCR Negative , N= 49 is what is written, please check the number , I think last digit is missing (?491)

Discussion - Some points on relevance of using case definitions for dengue surveillance can be added and how the improvement of the case definitions helps to improve the quality of surveillance.

Best wishes to the authors.
